# Supplementary material for: What Do We Know about Early Management of Sepsis and Septic Shock in Polish Hospitals? A Questionnaire Study
Source: Healthcare (Basel). 2021 Feb 1;9(2):140. doi: 10.3390/healthcare9020140 (PMC7912914; doi:10.3390/healthcare9020140)
Supplement: Supplementary file 1 [file healthcare-09-00140-s001.pdf]

Table S1. Differences in sepsis management between regions and within consecutive regions for major questions from the questionnaire

Therapeutic issue		Voivodeships	p[1]	p[2]	p[3]
Sepsis screening method	Clinical assessment	Greater Poland	0.47	0.24	0.047
		Silesia			0.07
		Masovia			0.10
	(quick) Sequential Organ Failure Score	Greater Poland	0.81	0.21	0.55
		Silesia			0.55
		Masovia			0.17
	National Early Warning Score 2	Greater Poland	0.53	0.49	<0.001
		Silesia			0.02
		Masovia			0.01
	Inflammatory markers	Greater Poland	0.41	0.33	0.04
		Silesia			0.45
		Masovia			0.25
Blood cultures taken when sepsis is suspected		Greater Poland	0.28	0.045	0.73
		Silesia			0.002
		Masovia			0.84
Hospital-based Standard Operating Procedures for antimicrobial treatment		Greater Poland	0.87	0.80	0.002
		Silesia			0.20
		Masovia			0.22
Prebiotics/ probiotics use		Greater Poland	0.40	0.29	0.63
		Silesia			0.59
		Masovia			0.02
Fluid responsiveness assessment	Clinical assessment	Greater Poland	0.73	0.73	0.35
		Silesia			0.002
		Masovia			0.02
	Arterial blood pressure	Greater Poland	0.41	0.24	0.046
		Silesia			0.28
		Masovia			0.04
	Diuresis	Greater Poland	0.17	0.54	0.12
		Silesia			0.81
		Masovia			0.19
	Lactate concentration	Greater Poland	0.10	0.33	0.51
		Silesia			0.64
		Masovia			0.58
	Dopamine	Greater Poland	0.15	0.13	0.16

		Silesia			0.044
		Masovia			0.19
Use of catecholamines	Dobutamine	Greater Poland	0.18	0.13	0.61
		Silesia			0.31
		Masovia			<0.001
	Epinephrine	Greater Poland	0.52	0.34	<0.001
		Silesia			0.03
		Masovia			0.03
	Norepinephrine	Greater Poland	0.97	0.96	0.049
		Silesia			0.06
		Masovia			0.04
	Argipressin	Greater Poland	0.52	0.59	0.11
		Silesia			<0.001
		Masovia			0.01
	Terlipressin	Greater Poland	0.56	0.55	0.02
		Silesia			0.45
		Masovia			0.001
Fluid therapy	Balanced crystalloids	Greater Poland	0.34	0.11	0.45
		Silesia			0.32
		Masovia			0.23
	Unbalanced crystalloids	Greater Poland	0.11	0.10	0.91
		Silesia			0.32
		Masovia			0.20
	Colloids (any)	Greater Poland	0.12	0.73	0.007
		Silesia			0.19
		Masovia			0.04
VICTAS protocol as an adjunct to hemodynamic support	Vitamin C	Greater Poland	0.44	0.11	0.048
		Silesia			0.57
		Masovia			0.04
	Thiamine	Greater Poland	0.15	0.79	0.71
		Silesia			0.03
		Masovia			0.65
	Steroids	Greater Poland	0.69	0.35	0.40
		Silesia			0.32
		Masovia			0.15
Extracorporeal blood purification techniques as an adjunct to hemodynamic support		Greater Poland	0.45	0.93	0.002
		Silesia			0.14
		Masovia			0.97

[1]Difference for the "in-ICU" management between voivodeships, [2]Difference for the "outside-ICU" management between voivodeships, [3]Difference Between "in-ICU" vs. "outside-ICU" management within particular voivodeship

Table S2: Elements of the Sepsis Care Bundles according to the Surviving Sepsis Campaign. [5,8,15,28]

Surviving Sepsis Campaign Sepsis Care Bundles

To be completed within 1 hour of time of presentation:

1. Measure lactate level*
2. Obtain blood cultures before administering antibiotics.
3. Administer broad-spectrum antibiotics.
4. Begin rapid administration of 30mL/kg crystalloid for hypotension or lactate level ≥ 4 mmol/L.
5. Apply vasopressors if hypotensive during or after fluid resuscitation to maintain MAP ≥ 65 mm Hg.

* Remeasure lactate if initial lactate is elevated (> 2 mmol/L)

To be completed within 3 hours of time of presentation:

1. Measure lactate level.
2. Obtain blood cultures before administration of antibiotics.
3. Administer broad-spectrum antibiotics.
4. Administer 30 mL/kg crystalloid for hypotension or lactate level greater than or equal to 4 mmol/L.

To be completed within 6 hours of time of presentation:

5. Apply vasopressors (for hypotension that does not respond to initial fluid resuscitation) to maintain an MAP greater than or equal to 65 mm Hg.
6. In the event of persistent hypotension after initial fluid administration (MAP <65 mm Hg) or if initial lactate level was greater than or equal to 4 mmol/L, reassess volume status and tissue perfusion
7. Remeasure lactate if initial lactate level is increased.
8. Document volume status and tissue perfusion changes with:

-Physical examination (after initial fluid resuscitation), including vital signs, capillary refill, pulse, and skin findings.
- Central venous catheter- central venous pressure and ScvO2 monitoring
- Bedside ultrasound examination
- Dynamic assessment of fluid responsiveness with passive leg raising test or fluid challenge

Time of presentation is defined as the time of triage in the ED or, if presenting from another care venue, from the earliest chart annotation consistent with all elements of severe sepsis or septic shock ascertained through chart review.

MAINTANANCE BUNDLES

1. Steroids, Thiamine, Vitamin C
2. Extracorporeal blood purification
3. Prebiotics/ Probiotics
4. Other Care Bundles for mechanical Ventilation, Sedation, early mobilization
